# An automated algorithm for the detection of cortical interruptions and its underlying loss of trabecular bone; a reproducibility study

**DOI:** 10.1186/s12880-018-0255-7

**Published:** 2018-05-15

**Authors:** M. Peters, J. de Jong, A. Scharmga, A. van Tubergen, P. Geusens, D. Loeffen, R. Weijers, S. K. Boyd, C. Barnabe, K. S. Stok, B. van Rietbergen, J. van den Bergh

**Affiliations:** 1 Department of Internal Medicine, Division of Rheumatology, Maastricht University Medical Centre, P.O. Box 5800, NL-6202 Maastricht, AZ the Netherlands; 2 CAPHRI, Care and Public Health Research Institute, Maastricht University, Maastricht, the Netherlands; 3 NUTRIM School for Nutrition and Translational Research in Metabolism, Maastricht University, Maastricht, the Netherlands; 4 Department of Radiology, Maastricht University Medical Centre, Maastricht, the Netherlands; 5 Faculty of Medicine and Life Sciences, Hasselt University, Hasselt, Belgium; 6 Cumming School of Medicine, McCaig Institute for Bone and Joint Health, University of Calgary, Calgary, Canada; 7 Department of Biomedical Engineering, the University of Melbourne, Melbourne, Australia; 8 Faculty of Biomedical Engineering, Eindhoven University of Technology, Eindhoven, the Netherlands; 9 Department of Orthopaedic Surgery, Maastricht University Medical Centre, Maastricht, the Netherlands; 10 Department of Internal Medicine, VieCuri Medical Centre, Venlo, the Netherlands

**Keywords:** Image processing, Precision, Cortical interruptions, Rheumatoid arthritis, High resolution peripheral quantitative computed tomography, Bone micro-architecture

## Abstract

**Ba*c*kground:**

We developed a semi-automated algorithm that detects cortical interruptions in finger joints using high-resolution peripheral quantitative computed tomography (HR-pQCT), and extended it with trabecular void volume measurement. In this study we tested the reproducibility of the algorithm using scan/re-scan data.

**Methods:**

Second and third metacarpophalangeal joints of 21 subjects (mean age 49 (SD 11) years, 17 early rheumatoid arthritis and 4 undifferentiated arthritis, all diagnosed < 1 year ago) were imaged twice by HR-pQCT on the same day with repositioning between scans. The images were analyzed twice by one operator (OP1) and once by an additional operator (OP2), who independently corrected the bone contours when necessary. The number, surface and volume of interruptions per joint were obtained. Intra- and inter-operator reliability and intra-operator reproducibility were determined by intra-class correlation coefficients (ICC). Intra-operator reproducibility errors were determined as the least significant change (LSC_SD_).

**Results:**

Per joint, the mean number of interruptions was 3.1 (SD 3.6), mean interruption surface 4.2 (SD 7.2) mm^2^, and mean interruption volume 3.5 (SD 10.6) mm^3^ for OP1. Intra- and inter-operator reliability was excellent for the cortical interruption parameters (ICC ≥0.91), except good for the inter-operator reliability of the interruption surface (ICC = 0.70). The LSC_SD_ per joint was 4.2 for the number of interruptions, 5.8 mm^2^ for interruption surface, and 3.2 mm^3^ for interruption volume.

**Conclusions:**

The algorithm was highly reproducible in the detection of cortical interruptions and their volume. Based on the LSC findings, the potential value of this algorithm for monitoring structural damage in the joints in early arthritis patients needs to be tested in clinical studies.

**Electronic supplementary material:**

The online version of this article (10.1186/s12880-018-0255-7) contains supplementary material, which is available to authorized users.

## Background

Rheumatoid arthritis (RA) is a chronic disease, in which inflammation at the joint may lead to erosions (i.e. cortical interruptions) [1, 2]. Interruptions in the cortical bone surface are often accompanied with underlying trabecular bone loss [3–5]. The presence, size and number of cortical interruptions within a joint, and the number of joints affected, are each associated with poor functional outcome and predictors of further progression of structural damage [3, 6, 7]. The quantification of interruptions on conventional radiography (CR) is considered the gold standard in clinical practice, however, it has a lower sensitivity compared to ultrasound, computed tomography (CT) and magnetic resonance imaging (MRI) [8–10].

High-resolution peripheral quantitative CT (HR-pQCT) is a low-dose imaging technique that is able to assess the three-dimensional (3D) bone structure at the micro-scale (82 μm nominal isotropic voxel size) of the peripheral skeleton in vivo [11]. Multiple studies have reported results on the visual inspection of the presence, number and size of interruptions with underlying trabecular bone voids in finger joints of patients with RA using HR-pQCT [12–22]. Excellent intra- and inter-rater reliability have been reported, but in all these studies only relatively large cortical interruptions were scored (mean diameter > 1.5 mm) [5, 13–15, 21, 23, 24]. In an earlier study, we showed that the inter-operator reliability is fair when visually scoring smaller cortical interruptions [25].

In addition, the quantification of interruption volume was primarily based on simple distance measures on a two-dimensional (2D) slice [5, 14, 15, 17, 18, 23]. A more extensive method is the 3D automated volume determination developed by Töpfer et al. [21]. However, in this method the location of the interruption still has to be visually identified by an operator. In addition, this volume determination was performed in large interruptions (average volume: 9.3mm^3^).

We have therefore developed a semi-automated algorithm that reliably detects small cortical interruptions (with a diameter ≥ 0.246 mm) [26]. In addition, we showed that interruptions with a diameter of ≥0.41 mm detected on HR-pQCT were also detected on μCT, the 3D gold standard [27]. However, this algorithm only analyzed the presence of an interruption in the cortex and did not consider the underlying trabecular bone loss as part of the total interruption volume. This is important because not only the presence but also the size of cortical interruptions (which includes the trabecular bone void) are associated with poor functional outcome and predictors of further progression of structural damage [3, 6, 7].

Furthermore, the reproducibility of our algorithm on scan/re-scan with repositioning in-between the scans has not yet been tested in the standard workflow of the HR-pQCT scanner. Only the effect of the operator was investigated and not the influence of re-positioning of the hand nor the effect of image quality (noise and motion artifacts) in addition to the effect of the operator. Two previous studies tested the reproducibility on scan/re-scan data in the standard workflow of the HR-pQCT scanner for structural and density parameters in metacarpal heads [13, 28]. The density parameters showed precision errors of ≤2%, but for trabecular and cortical structural parameters, precision errors up to 33% were found [13, 28]. However, precision errors of the cortical parameters were only tested in healthy controls and not in early arthritis patients. Moreover, the phalangeal base was not included as part of the metacarpophalangeal (MCP) joint.

Therefore, the aims of this study were: 1) to extend our algorithm for detection of cortical interruptions with underlying trabecular bone void volume detection, 2) to evaluate the precision errors of our algorithm to detect cortical interruptions and its volume using scan/re-scan data in the standard workflow of the HR-pQCT scanner, and 3) to evaluate the precision errors of the trabecular and cortical density and micro-structure parameters in the standard workflow of the HR-pQCT scanner.

## Methods

### Patient population

Twenty-one patients were recruited (mean age 49 (SD 11) years) with early RA (*n* = 17) and undifferentiated arthritis (*n* = 4), all diagnosed < 1 year from the Early Inflammatory Arthritis Clinics of the Division of Rheumatology at the University of Calgary, Canada. All patients with early RA fulfilled the 2010 American College of Rheumatology (ACR)/European League Against Rheumatism (EULAR) classification criteria for RA [29]. Ethical approval was obtained from the Conjoint Health Research Ethics Board at the University of Calgary, Canada (REB 15–0582). All participants signed informed consent.

### HR-pQCT scanning procedure

The second and third metacarpophalangeal joints of the dominant hand were scanned twice with HR-pQCT (XtremeCT, Scanco Medical AG, Switzerland) using the image acquisition protocol developed by the Study group for xtrEme Computed Tomography in RA (SPECTRA), an international collaboration of HR-pQCT users [30]. Scanning was performed at clinical in vivo settings, i.e. at 60kVp tube voltage, 900 μA tube current, 100 ms integration time and images were reconstructed using an 82 μm nominal isotropic voxel size. The reference line was placed at the midpoint of the concave articular surface at the base of the second or third proximal phalanx (whichever was the most distal of the two). The scan covered a length of 9.02 mm (1 stack) in the distal direction and 18.04 mm (2 stacks) in the proximal direction (total scan length 27.06 mm, 330 slices, 3 stacks) (Additional file 1). The total scanning time was approximately 9 min. After the first scan, the patients removed their hand from the stabilization platform, rested for five minutes, and were then repositioned for a second scan. Subject scans were evaluated on motion artifacts per stack according to Pialat et al. [31]. Stacks of poor quality (grade > 3) on the first and/or second scan were excluded from further analyses [31].

### Image analysis

#### Outer contour

**Fig. 1:**
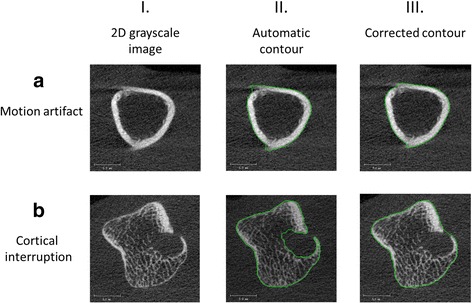
Typical examples of 2D grayscale images in which the contour is manually corrected by the operators. **a** Due to a motion artifact, the automatically obtained contour was not tight around its original structure (**a**. II). The operators corrected this (**a**. III). In (**b**) a large cortical interruption with underlying trabecular bone loss is shown (**b**. I, arrow). The automatically obtained contour does not follow the outer margin of the original structure at the cortical interruption (**b**. II). The operators therefore corrected the contour (**b**. III)

The outer margin of the cortex was identified using a modified previously described auto-contouring algorithm for periosteal segmentation of the distal radius and tibia [26]. For the first scans, the contours were visually inspected and, if necessary, corrected by one operator twice (MP, OP1) with three years of experience with HR-pQCT to enable calculation of intra-operator reliability. Contouring of the first scans was performed by one additional operator (JdJ, OP2) with five years of experience with HR-pQCT to enable calculation of inter-operator reliability. For the second scans, the contours were corrected by OP1, to enable calculation of intra-operator reproducibility. The number of manual corrections largely depends on the presence and severity of motion artifacts and the number of large cortical interruptions (>1mm^2^) within the joint. Corrections are always necessary in case of large cortical interruptions and usually necessary with motion grades ≥3. Figure 1 shows two examples of contours that were corrected by the operators, one due to motion artifacts (Fig. 1a), and another due to a large cortical interruption (Fig. 1b).

#### Binary image, and bone density and micro-structure parameters

The outer contours obtained from OP1 and OP2 were used for standard and cortical evaluation protocols [11, 32]. The cortical evaluation protocol incorporates the detection of the inner cortical contour as explained by Burghardt et al. [32]. No corrections were applied to the inner cortical contour. The standard evaluation protocol from the manufacturer (Scanco Medical AG, Bruttisellen, Switzerland) for radius and tibia, which included Laplace-Hamming filtering and thresholding [33], was used to distinguish bone from non-bone voxels to create a 3D binary image, and to determine the bone density and micro-architectural parameters as described elsewhere [11]. From the standard evaluation protocol, the volumetric bone mineral density (vBMD) [mg HA/cm^3^] in the total (Tot.BMD) and trabecular (Tb.BMD) region was obtained [11]. Furthermore, trabecular number (Tb.N) [mm^− 1^], thickness (Tb.Th) [μm], separation (Tb.Sp) [μm], and intra-individual distribution of separation (Tb.SpSD) [μm] were determined to assess the trabecular compartment [11]. From the cortical evaluation protocol, the cortical density (Ct.BMD) and the density of the cortical bone tissue (Ct.TMD) were obtained. The cortical thickness (Ct.Th) [μm], cortical porosity (Ct.Po) [%], and cortical pore diameter (Ct.Po.Dm) [μm] were determined to assess the cortical compartment [32].

#### Cortical interruption detection algorithm

The algorithm is developed within the scanner software (Image Processing Language (IPL)). The first part of the algorithm has been described in detail elsewhere [26, 27]. In short, first, a cortical mask with a constant depth of 4 voxels (0.328 mm) is generated in 3D based on the outer contour. Second, the cortical bone within the masked region is analyzed for discontinuities that can be considered cortical interruptions (diameter ≥ 0.41 mm), because these were also detected on μCT scans [27].

**Fig. 2:**
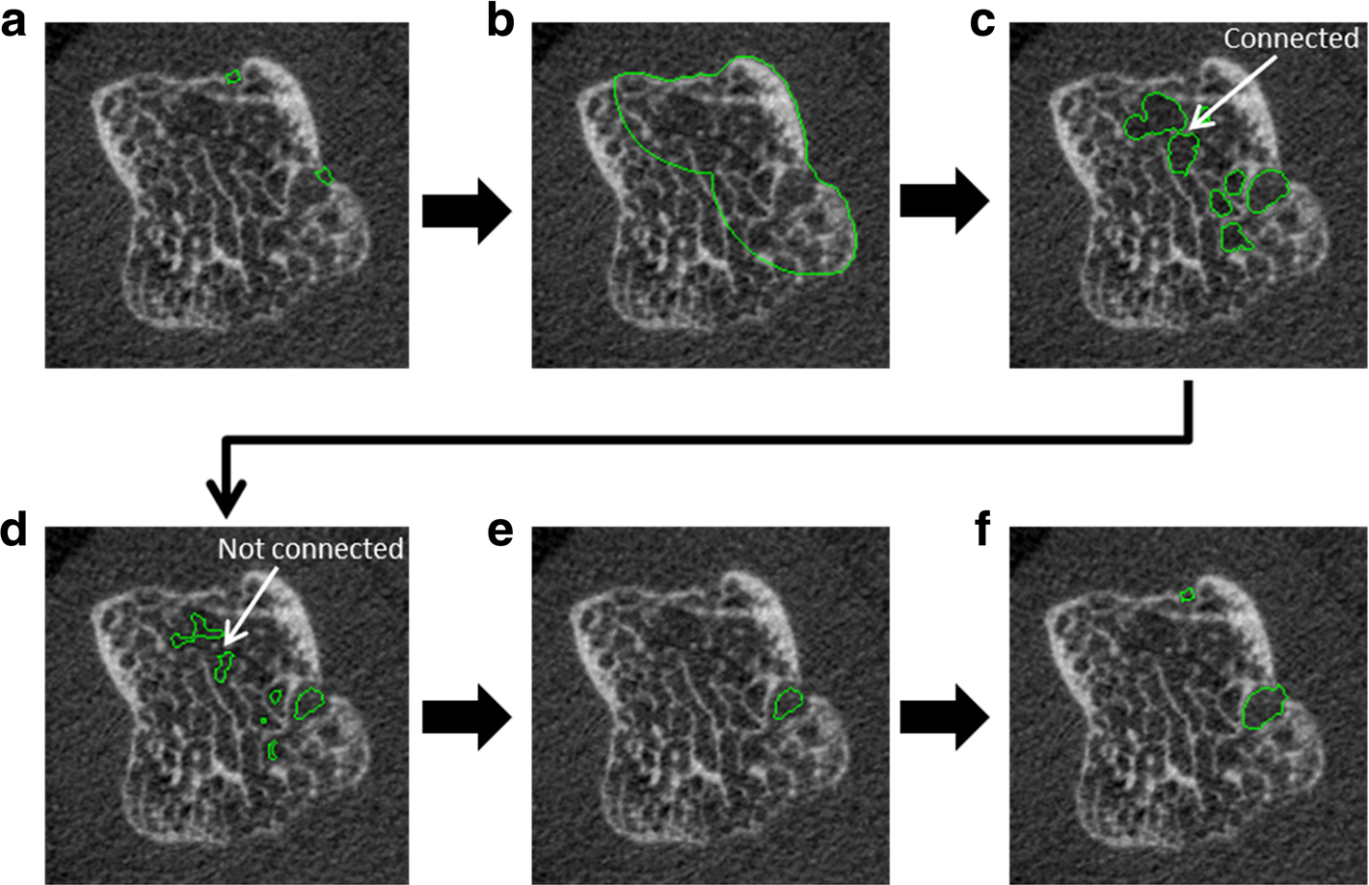
Representation of the steps executed by the extended part of the algorithm. **a** Two cortical interruptions ≥0.41 mm are visualized on a 2D grayscale image as output of the first part of the algorithm. A region of interest (ROI) is obtained by dilating the detected cortical interruptions with 48 voxels (=3.936 mm) and masked with the outer contour (**b**). Within this ROI, a distance transformation is performed. Only voids that are ≥0.738 mm in diameter are selected (**c**). These volumes are eroded by 2 voxels to lose connections of ≤0.328 mm and therefore prevent leaking into the trabecular structure (**d**). The voids that remain connected to a cortical interruption are included (**e**), dilated to its original size and the originally detected cortical interruptions are added (**f**)

The extended part of the algorithm is explained in Fig. 2. As an example, two cortical interruptions ≥0.41 mm are visualized on a 2D grayscale image as output of the first part of the algorithm (Fig. 2a, green circles). A region of interest (ROI) is obtained by dilating the detected cortical interruptions 48 times (= 3.936 mm) in all three dimensions, which results in a sphere with a radius of 4 mm. This contour was masked with the outer contour to only consider the region within the bone (Fig. 2b). A radius of 4 mm was chosen because it approximates half the width of the metacarpal head (Fig. 2b). The ROI prevents connection of detected voids with the intramedullary canal void. Within this ROI, a 3D distance transformation is performed, and only trabecular voids that are ≥0.738 mm in diameter are selected (Fig. 2c), which is higher than trabecular separation commonly (< 5%) observed in MCP joints of healthy controls [13].

The detected voids are then eroded by 2 voxels in all three dimensions, which leads to loss of connections of ≤0.328 mm in diameter, and therefore prevents leaking of the voids into the trabecular structure (Fig. 2d). Only those voids that remain connected to a cortical interruption are included (Fig. 2e). Last, these selected voids are dilated in all three dimensions to their original size and the originally detected cortical interruptions are added to these voids (Fig. 2f).

Per joint, the number of cortical interruptions detected, interruption surface, and interruption volume (cortical interruption + trabecular void volume) were obtained.

### Statistical analysis

Descriptive statistics per joint were calculated for the number of interruptions, interruption surface and volume, and bone density and micro-structure parameters. Paired t-test was used to compare the results per joint between the first and second scan assessments. Intra- and inter-operator reliability and intra-operator reproducibility on the joint level was assessed by intra-class correlation coefficient (ICC) with a two-way random model and absolute agreement. ICCs were rated as: < 0.40 poor, 0.40–0.60 fair, 0.60–0.75 good, and 0.75–1.00 excellent. In addition, intra-operator reliability and reproducibility errors were determined as the root mean square (RMS) of coefficients of variation (CV) and the RMS of the standard deviation (SD), respectively CV_RMS_ and SD_RMS_, as described by Glüer et al. [34]. The Least Significant Change (LSC) was calculated in absolute values (LSC_SD_) and in percentages (LSC_CV%_) according to eqs. 1 and 2. Bland-Altman plots were made for qualitative assessment of the intra- and inter-operator reliability, and intra-operator reproducibility for all parameters. Statistical analyses were performed using IBM SPSS Statistics for Windows, Version 20.0 (IBM Corp., Armonk, NY).

1 $$ {LSC}_{SD}\kern1.25em =1.96\ast \sqrt{2}\ast {SD}_{RMS} $$

2 $$ {LSC}_{CV\%}\kern0.5em =1.96\ast \sqrt{2}\ast {CV}_{RMS} $$

## Results

**Fig. 3:**
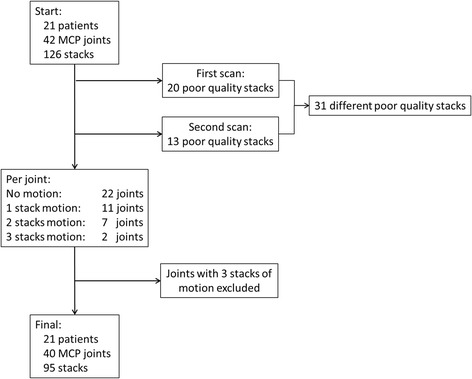
Schematic overview of the exclusion of joints due to motion artifacts

In 42 different patient scans with 126 stacks, 20 (15.9%) stacks of poor quality were observed at the first scan and 13 (10.3%) at the second scan. Two stacks were of poor quality at both the first and second scan. Therefore, in total 31 out of 126 stacks (24.6%) were excluded from the analysis due to motion artifacts. Two joints, which had poor quality on all three stacks, were excluded (Fig. 3). Hence, 40 joints remained for analysis (Fig. 3).

### Visual assessment

**Fig. 4:**
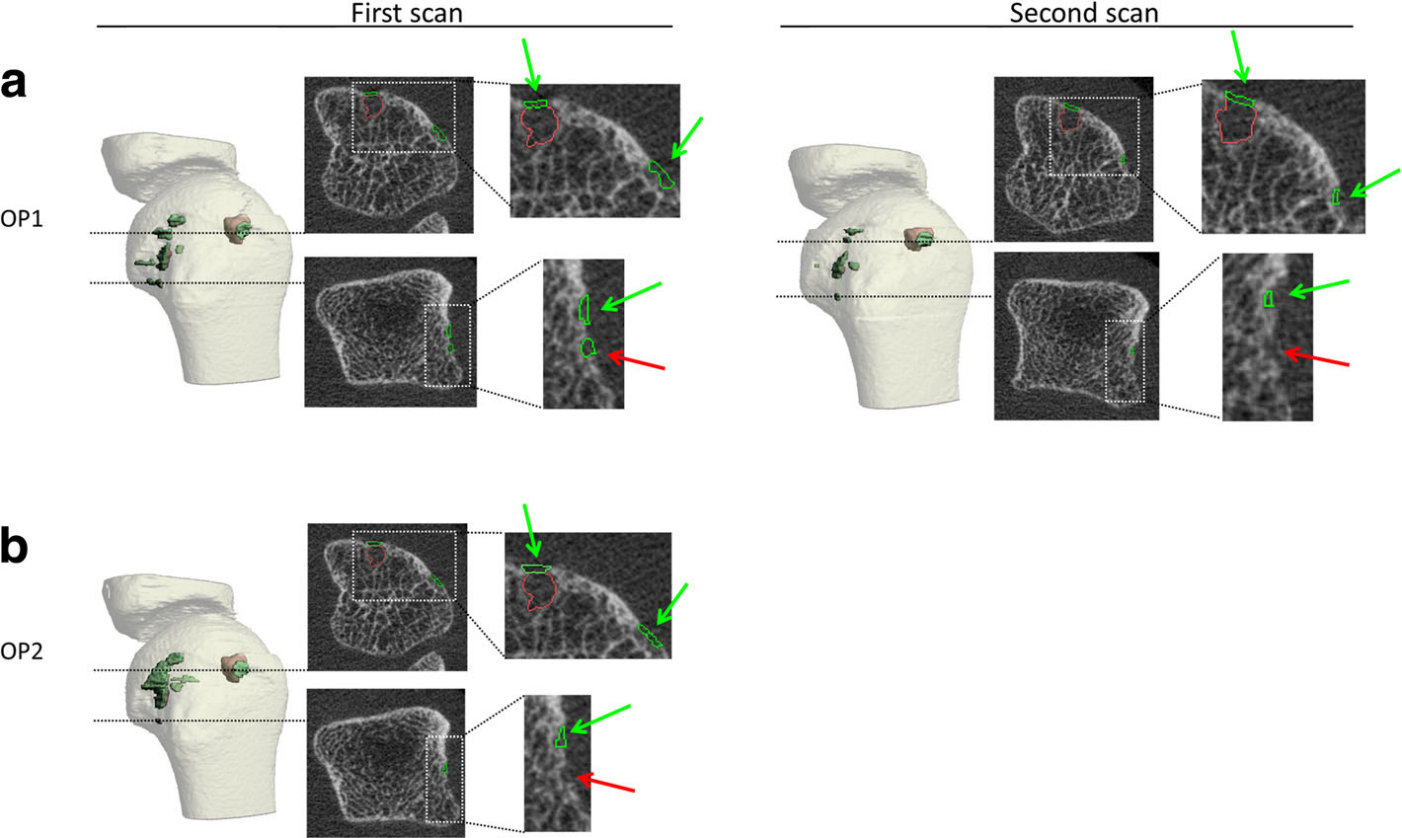
Visual outputs of the algorithm in 3D and 2D of an MCP joint. The outputs of OP1 (**a**.) and OP2 (**b**.) at the first and second scan performed are shown. Shown are the 3D outputs with multiple detected cortical interruptions (green) and underlying trabecular bone voids (red) with corresponding 2D grayscale images. The algorithm indeed accurately fills the underlying trabecular bone voids, and it can be seen that most interruptions are detected at the same location on the first and second scan, and by both OP1 and OP2 (green arrows). However, some discrepancies were also seen (red arrows)

Cortical interruptions detected by the algorithm based on the corrected contours of OP1 and OP2 on the first scan and corrected contours of OP1 on the second scan are visualized in 3D, and visualized on corresponding 2D grayscale images (Fig. 4). Fig. 4a shows that the algorithm accurately detects cortical interruptions (green) and its underlying trabecular void volume (red), and that most interruptions were detected on both the first and second scan (green arrows). However, discrepancies were also found (red arrow).

Similarly, most interruptions detected by the algorithm on the first scan using the corrected contours of OP1 were also found when using the corrected contours of OP2 (green arrows), but discrepancies were found as well (red arrow).

### Quantitative assessment

#### Reliability

Intra- and inter-operator reliability was excellent for all bone density and micro-structure parameters (ICC > 0.99). Bland-Altman plots show that no cut-off bias was observed for all bone density and micro-structure parameters, and that the errors were independent of the mean values detected (Additional files 2 and 3). Intra- and inter-operator reliability of the cortical interruption algorithm was excellent for the cortical interruption parameters (ICCs ≥0.91), except for the inter-operator reliability of the interruption surface (ICC = 0.70, Additional file 4). For the intra-operator reliability, LSC_SD_ values were 2.0 for the number of interruptions, 4.6 mm^2^ for the interruption surface, and 1.9 mm^3^ for the interruption volume (Additional file 4). Bland-Altman plots show no cut-off bias and the errors were independent of the mean values detected (Additional file 5a). For the inter-operator reliability, Bland-Altman plots show no cut-off bias for the number of interruptions and the errors were independent of the number of interruptions detected (Additional file 5b). However, for the interruption surface and volume, OP2 had higher outcomes compared to OP1, and this increased with increasing mean value (Additional file 3b).

#### Reproducibility

Table 1 shows the results for the bone density and micro-structure parameters, and the number of interruptions, interruption surface and interruption volume detected by OP1 on the first and second scan. No statistical difference was found for all outcomes between the first and second scan.

**Table 1:** Reproducibility of the cortical interruption parameters, and bone density and micro-structure parameters

		Mean (SD)	ICC (95% CI)	SD_RMS_	LSC_SD_	CV_RMS_	LSC_CV%_
Cortical interruption parameters							
Number of interruptions		3.1 (3.4)	0.82 (0.69 - 0.90)	1.5	4.2	n.a.	n.a.
Interruption surface	mm²	4.2 (7.1)	0.92 (0.85 - 0.96)	2.1	5.8	n.a.	n.a.
Interruption volume	mm³	3.5 (10.7)	0.99 (0.98 - 0.99)	1.1	3.2	n.a.	n.a.
Bone density parameters							
Tot.BMD	mg HA/cm³	341.8 (72.2)	1.00 (1.00 - 1.00)	3.8	10.6	1.1	3.2
Tb.BMD	mg HA/cm³	163.4 (47.2)	0.99 (0.99 - 1.00)	3.6	10.0	2.6	7.3
Ct.BMD	mg HA/cm³	883.7 (94.5)	1.00 (1.00 - 1.00)	4.5	12.4	0.5	1.4
Ct.TMD	mg HA/cm³	939.8 (70.6)	1.00 (1.00 - 1.00)	3.3	9.1	0.4	1.0
Bone micro-structure parameters							
Tb.N	mmˉ¹	1.56 (0.49)	0.99 (0.98 - 0.99)	0.06	0.16	4.2	11.7
Tb.Th	μm	90.7 (22.7)	0.98 (0.95 - 0.99)	3.6	10.1	4.3	11.8
Tb.Sp	μm	630.4 (259.5)	0.98 (0.96 - 0.99)	39.6	109.7	4.4	12.1
Tb.SpSD	μm	558.3 (316.2)	0.97 (0.95 - 0.99)	54.0	149.7	7.7	21.3
Ct.Th	μm	889.7 (196.2)	1.00 (0.99 - 1.00)	15.1	41.8	1.4	3.9
Ct.Po	%	4.31 (3.07)	1.00 (0.99 - 1.00)	0.35	0.96	8.7	24.1
Ct.Po.Dm	μm	197.3 (86.3)	0.84 (0.72 - 0.91)	36.7	101.7	7.6	21.1

Values are displayed as mean (SD), and for ICC as value (95% Confidence Interval)

*n.a.* not applicable; CV_RMS_ and LSC_CV%_ were only determined for continuous variables

*ICC* intra-class correlation coefficient, *SD*_*RMS*_ root mean square of the standard deviation, *LSC*_*SD*_ absolute least significant change, *CV*_*RMS*_ root mean square of the coefficient of variation, *LSC*_*CV%*_ least significant change in percentages, *Tot.BMD* total volumetric bone mineral density, *Tb.BMD* trabecular BMD, *Ct.BMD* cortical BMD, *Ct.TMD* cortical bone tissue BMD, *Tb.N* trabecular number, *Tb.Th* trabecular thickness, *Tb.Sp* trabecular separation, *Tb.SpSD* intra-individual distribution of trabecular separation, *Ct.Th* cortical thickness, *Ct.Po* cortical porosity, *Ct.Po.Dm* cortical porosity diameter

The reproducibility for the bone density and micro-structure parameters was excellent with ICCs ≥0.84 (Table 1). The precision errors in percentages (CV_RMS_) were generally < 5%, except for Tb.SpSD, Ct.Po and Ct.PoDm (CV_RMS_ = 7.7, 8.7 and 7.6%, respectively). The LSC_CV%_ was ≤7.3% for the bone density parameters, but up to 24.1% for the bone micro-structure parameters. Bland-Altman plots show that for the intra-operator reproducibility no cut-off bias was observed for all bone density and micro-structure parameters, and that the errors were independent of the mean values detected (Additional file 6).

Reproducibility of the cortical interruption algorithm was also excellent for all outcomes with ICCs ≥0.82 (Table 1), especially for the interruption volume (ICC 0.99, Table 1). The precision errors SD_RMS_ per joint was 1.5 for the number of interruptions, 2.1 mm^2^ for the interruption surface, and 1.1 mm^3^ for the interruption volume. The LSC_SD_ was 4.2 for the number of interruptions, 5.8 mm^2^ for interruption surface, and 3.2 mm^3^ for the interruption volume. Bland-Altman plots show that for the intra-operator reproducibility no cut-off bias was observed for the number, surface and volume of interruptions, and that the errors were independent of the mean values detected (Additional file 5c).

## Discussion

In this study, we calculated the precision errors of our extended algorithm for detection of cortical interruptions and underlying trabecular bone void volume in MCP joints on scan/re-scan HR-pQCT data with repositioning in-between the scans in early arthritis patients. In addition, we calculated the precision errors for the bone density and micro-structure parameters. Reproducibility of our algorithm was excellent (ICCs ≥0.82), especially for the interruption volume (ICC 0.99). Reproducibility for the bone density and micro-structure parameters was also excellent (ICCs ≥0.84). Bland-Altman plots showed no systematic error in the reproducibility of our algorithm, bone density and bone micro-structure parameters. The reproducibility LSC_SD_ value per joint was 4.2 for number of interruptions, 5.8 mm^2^ for interruption surface, and 3.2 mm^3^ for interruption volume.

The intra-operator reproducibility LSC_SD_ value of the algorithm for the interruption volume was higher in our study than the intra- and inter-operator LSC_SD_ reported by Töpfer et al. for single interruptions (LSC 3.2 mm^3^ versus 1.4 mm^3^ and 2.1 mm^3^, respectively) [21]. The study from Töpfer et al. differed in several aspects from ours. They analyzed a selection of interruptions in one dataset on its volume by two operators. In contrast, we used scan/re-scan data and included all interruptions, irrespective whether they were detected on the first scan but not on the second scan and vice versa. These aspects will lead to higher reproducibility errors. By excluding the effect of the rescanning (i.e. intra-operator reliability), the LSC_SD_ value was comparable to the study of Töpfer et al. (LSC 1.9mm^3^ versus 1.4mm^3^) [21].

In our study, we also included the phalangeal base, thus creating a larger scan region for analyzing bone density and micro-structure parameters. This did not affect the precision errors, except for Ct.Po, which was substantially lower compared to a previous study (8.7% versus 27.7%) [28]. The precision errors of the other parameters obtained in our study were similar as in previous studies [13, 28]. In our study, the precision errors (CV_RMS_) were generally below 2% for the bone density parameters (except for Tb.BMD), below 5% for the trabecular bone parameters (except for Tb.SpSD), below 10% for the cortical bone parameters. The precision errors are also comparable as observed in radius and tibia scans [11].

The mean number of cortical interruptions and interruption surface per joint detected in this study (3.1 and 4.2 mm^2^, respectively) were substantially lower than in our previously reported study using the same algorithm (9.5 and 13.5 mm^2^, respectively) [27]. In our previous study, anatomic specimens from high-aged subjects (mean 85.1 years) were used with a low BMD (vBMD of the joints: 245 mgHA/cm^3^ versus 338 mgHA/cm^3^ in this study). In the present study, the bone is better mineralized and therefore these voxels are less likely to represent non-bone voxels after segmentation and, hence, a lower number of interruptions was found.

The mean volume of the interruptions detected with the algorithm was substantially lower compared to previous studies that investigated volumes of interruptions in 3D (1.1 mm^3^ versus > 4 mm^3^), confirming that our algorithm enables the detection of much smaller interruptions [13, 21, 24].

Our study has several limitations. First, with our algorithm, the trabecular void volume underlying the cortical interruption that can be detected is limited to a depth of 4 mm. This means that the algorithm underestimates the volume of interruptions with a depth greater than 4 mm. However, 4 mm is approximately half the width of the metacarpal head. Hence, such interruptions are not the primary focus of research with HR-pQCT, because these large interruptions can also be detected by other imaging techniques with lower resolution. For example, “small” interruptions, i.e. < 10 mm^3^ are occasionally missed with MRI [12]. Thus, our algorithm can best be used for studies with HR-pQCT aiming at early detection of structural damage, i.e. small interruptions, in patients with RA. Second, our algorithm requires manual correction of the outer margin of the contours in case of large cortical interruptions and motion artifacts which can make the analysis time consuming [26]. However, this correction is also advised by the manufacturer for the standard evaluation protocol for assessment of the bone density and micro-structure parameters. Further automation of the outer contour would improve the applicability. The strength of our algorithm is that it is developed within the scanner software (IPL). Therefore, the algorithm can be easily implemented to other scanners.

The current investigation of the reproducibility of the algorithm and the extension of underlying trabecular bone void detection was a next step in the validation of our algorithm in the detection of small cortical interruptions in finger joints by HR-pQCT. We found that the algorithm was highly reproducible, but still had substantial precision errors compared to the mean value detected. Therefore, the next step is to test this algorithm in clinical studies in order to determine its potential value in monitoring patients with RA, and discriminating patients with RA, preferably early in the disease course, from healthy controls.

## Conclusions

The extended algorithm for detection of cortical interruptions and their volume, and the assessment of the bone density and micro-structure parameters on HR-pQCT is highly reproducible in finger joints of early arthritis patients. The potential value of this algorithm for monitoring structural damage in the joints in early arthritis patients needs to be tested in clinical studies.

## Additional files

Additional file 1: Scout view of the right hand, showing the region that was scanned by the HR-pQCT during both scans. The proximal edge of the phalangeal base of the most distal joint (MCP2 in this case) was chosen as the landmark for the placement of the reference line. The scan region was 27.06 mm (3 stacks) long with 9.02 mm (1 stack) distal of the reference line and 18.04 mm (2 stacks) proximal of the reference line. (TIF 1765 kb)

Additional file 2: Bland-Altman plots of the intra-operator reliability for the bone density and bone micro-structural parameters. Bland-Altman plots for all bone density (A), trabecular micro-structure (B) and cortical micro-structure (C) parameters for the intra-operator reliability. For all parameters, no cut-off bias was observed and the errors were independent of the mean values detected. BMD, volumetric bone mineral density; Tot.BMD, total BMD; Tb.BMD, trabecular BMD; Ct.BMD, cortical BMD; Ct.TMD, cortical bone tissue BMD; Tb.N, trabecular number; Tb.Th, trabecular thickness; Tb.Sp, trabecular separation; Tb.SpSD, intra-individual distribution of trabecular separation; Ct.Th, cortical thickness; Ct.Po, cortical porosity; Ct.Po.Dm, cortical porosity diameter (TIF 949 kb)

Additional file 3: Bland-Altman plots of the inter-operator reliability for the bone density and bone micro-structural parameters. Bland-Altman plots for all bone density (A), trabecular micro-structure (B) and cortical micro-structure (C) parameters for the inter-operator reliability. For all parameters, no cut-off bias was observed and the errors were independent of the mean values detected. BMD, volumetric bone mineral density; Tot.BMD, total BMD; Tb.BMD, trabecular BMD; Ct.BMD, cortical BMD; Ct.TMD, cortical bone tissue BMD; Tb.N, trabecular number; Tb.Th, trabecular thickness; Tb.Sp, trabecular separation; Tb.SpSD, intra-individual distribution of trabecular separation; Ct.Th, cortical thickness; Ct.Po, cortical porosity; Ct.Po.Dm, cortical porosity diameter (TIF 950 kb)

Additional file 4: Intra- and inter-operator reliability of the cortical interruption parameters. Table with the intra- and inter-operator reliability of the cortical interruption parameters. Values are as value (95% Confidence Interval) ICC, intra-class correlation coefficient; SD_RMS_, root mean square of the standard deviation; LSC_SD_, absolute least significant change (DOCX 20 kb)

Additional file 5: Bland-Altman plots of the intra- and inter-operator reliability and intra-operator reproducibility for all cortical interruption parameters. Bland-Altman plots for all cortical interruption parameters for the intra- operator reliability (A), inter-operator reliability (B) and intra-operator reproducibility (C). (A) For the intra-operator reliability, no cut-off bias was observed for the number, surface and volume of interruptions and the errors were independent of the mean values detected. (B) For the inter-operator reliability, no cut-off bias was observed for the number of interruptions and the errors were independent of the number of interruptions detected. For the interruption surface and volume, OP2 had higher outcomes compared to operator 1, and this increased with increasing mean value. (C) For the intra-operator reproducibility, no cut-off bias was observed for the number, surface and volume of interruptions and the errors were independent of the mean values detected (TIF 971 kb)

Additional file 6: Bland-Altman plots of the intra-operator reproducibility for the bone density and bone micro-structural parameters. Bland-Altman plots for all bone density (A), trabecular micro-structure (B) and cortical micro-structure (C) parameters for the intra-operator reproducibility. For all parameters, no cut-off bias was observed and the errors were independent of the mean values detected. BMD, volumetric bone mineral density; Tot.BMD, total BMD; Tb.BMD, trabecular BMD; Ct.BMD, cortical BMD; Ct.TMD, cortical bone tissue BMD; Tb.N, trabecular number; Tb.Th, trabecular thickness; Tb.Sp, trabecular separation; Tb.SpSD, intra-individual distribution of trabecular separation; Ct.Th, cortical thickness; Ct.Po, cortical porosity; Ct.Po.Dm, cortical porosity diameter (TIF 1028 kb)

Additional file 7: Data of the cortical interruption-, bone density- and micro-structure parameters. Data of the cortical interruption parameters, and bone density and micro-structure parameters on the first (OP1 twice and OP2) and second scan (OP1). (XLS 63 kb)

## Abbreviations

2D: Two-dimensional
3D: Three-dimensional
ACR: American College of Rheumatology
CR: Conventional radiography
CT: Computed tomography
Ct.BM: Cortical BMD
Ct.Po: Cortical porosity
Ct.Po.Dm: Cortical pore diameter
Ct.Th: Cortical thickness
Ct.TMD: Cortical tissue mineral density
CV: Coefficients of variation
EULAR: European League Against Rheumatism
HR-pQC: High-resolution peripheral quantitative computed tomography
ICC: Intra-class correlation coefficient
IPL: Image processing language
LSC: Least significant change
MCP: Metacarpophalangeal
MRI: Magnetic resonance imaging
OP1: Operator 1
OP2: Operator 2
RA: Rheumatoid arthritis
RMS: Root mean square
SD: Standard deviation
SPECTRA: Study grouP for xtrEme Computed Tomography in RA
Tb.BMD: Trabecular BMD
Tb.N: Trabecular number
Tb.Sp: Trabecular separation
Tb.SpSD: Distribution of trabecular separation
Tb.Th: Trabecular thickness
Tot.BMD: Total BMD
vBMD: Volumetric bone mineral density
