# Supplementary figures and images for: An automated algorithm for the detection of cortical interruptions and its underlying loss of trabecular bone; a reproducibility study
Source: BMC Med Imaging. 2018 May 15;18:13. doi: 10.1186/s12880-018-0255-7 (PMC5952860; doi:10.1186/s12880-018-0255-7)

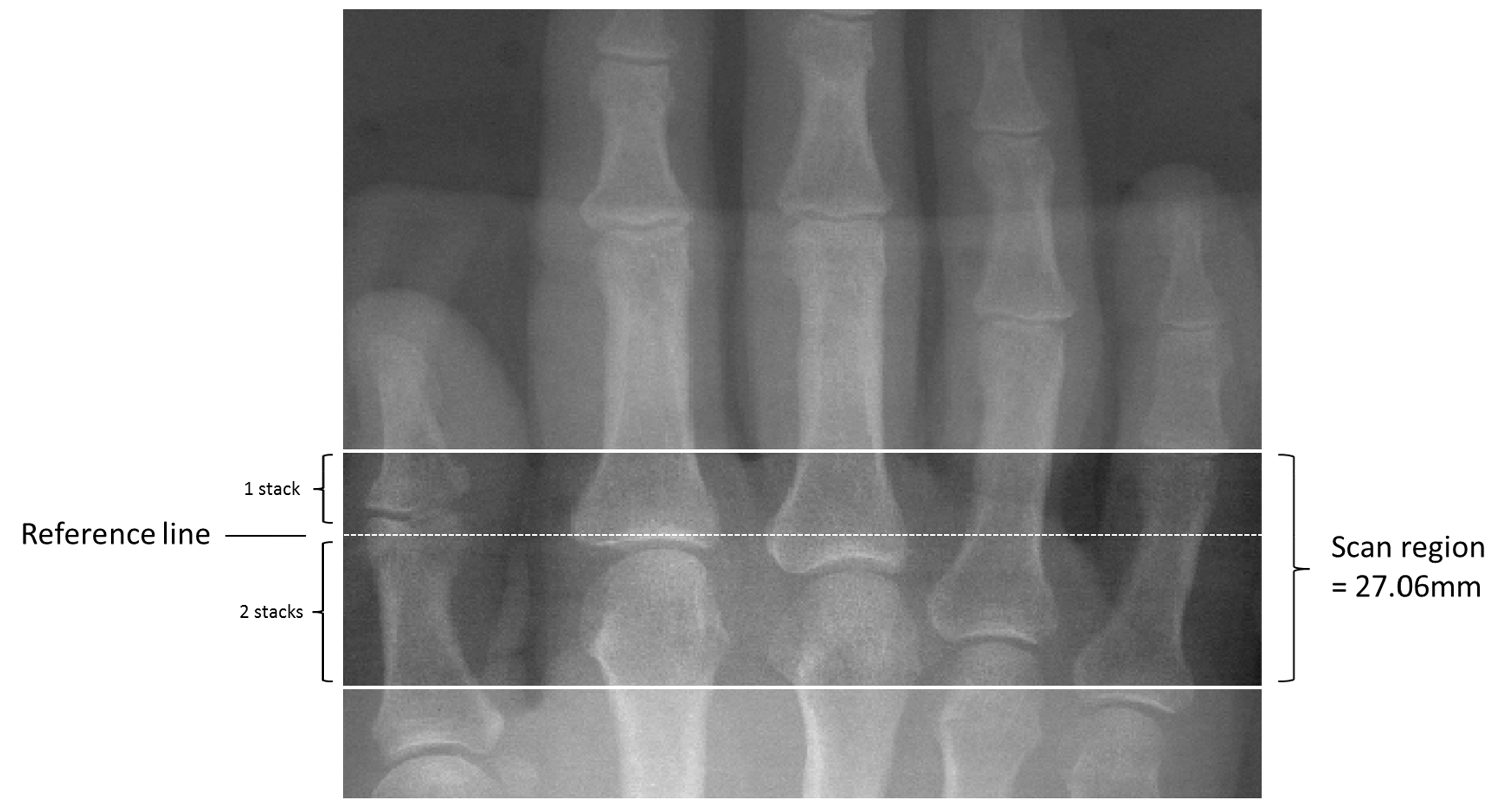

Supplement: Supplementary file 1 — Scout view of the right hand, showing the region that was scanned by the HR-pQCT during both scans. The proximal edge of the phalangeal base of the most distal joint (MCP2 in this case) was chosen as the landmark for the placement of the reference line. The scan region was 27.06 mm (3 stacks) long with 9.02 mm (1 stack) distal of the reference line and 18.04 mm (2 stacks) proximal of the reference line. (TIF 1765 kb) [file 12880_2018_255_MOESM1_ESM.tif]

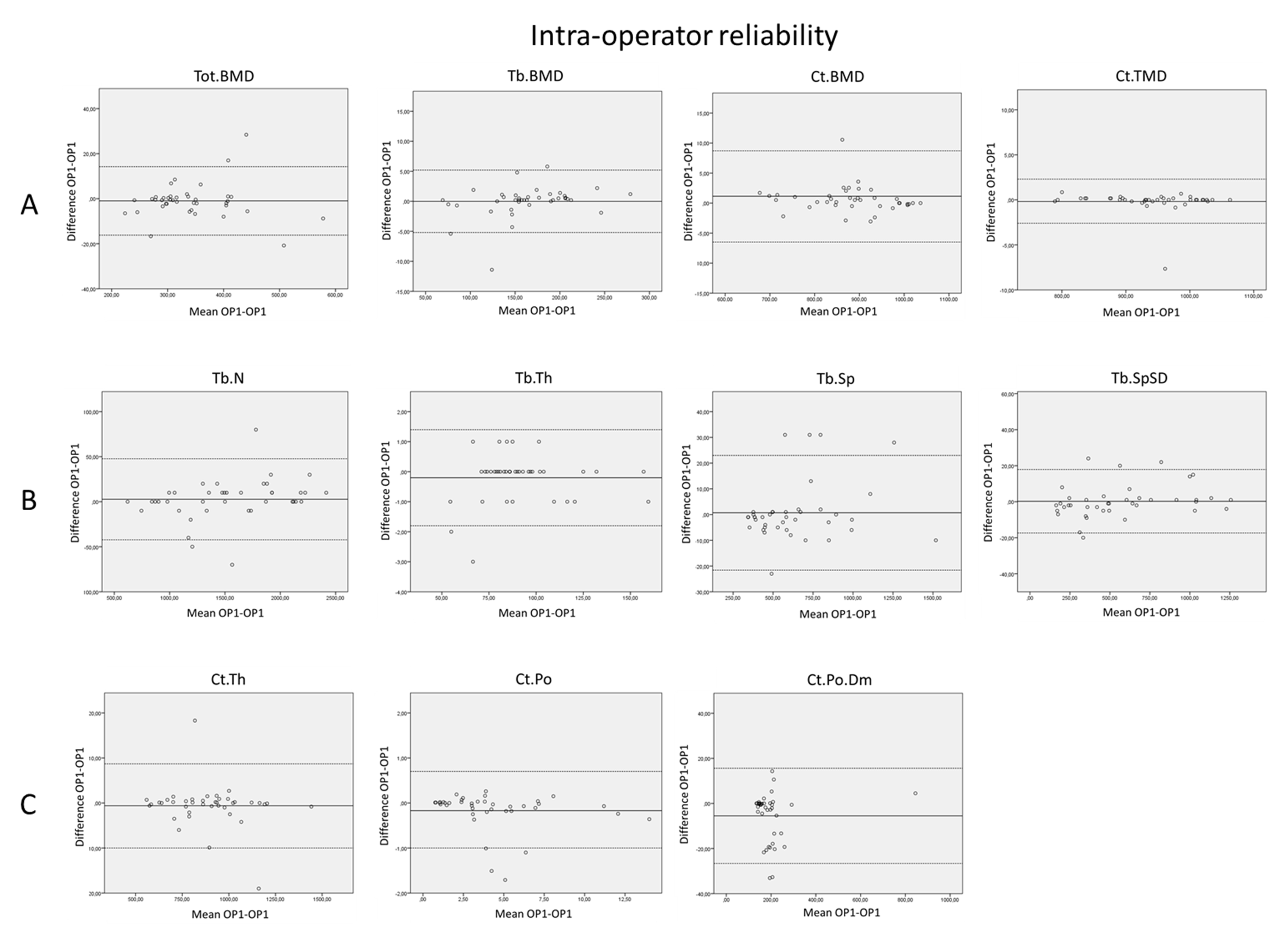

Supplement: Supplementary file 2 — Bland-Altman plots of the intra-operator reliability for the bone density and bone micro-structural parameters. Bland-Altman plots for all bone density (A), trabecular micro-structure (B) and cortical micro-structure (C) parameters for the intra-operator reliability. For all parameters, no cut-off bias was observed and the errors were independent of the mean values detected. BMD, volumetric bone mineral density; Tot.BMD, total BMD; Tb.BMD, trabecular BMD; Ct.BMD, cortical BMD; Ct.TMD, cortical bone tissue BMD; Tb.N, trabecular number; Tb.Th, trabecular thickness; Tb.Sp, trabecular separation; Tb.SpSD, intra-individual distribution of trabecular separation; Ct.Th, cortical thickness; Ct.Po, cortical porosity; Ct.Po.Dm, cortical porosity diameter (TIF 949 kb) [file 12880_2018_255_MOESM2_ESM.tif]

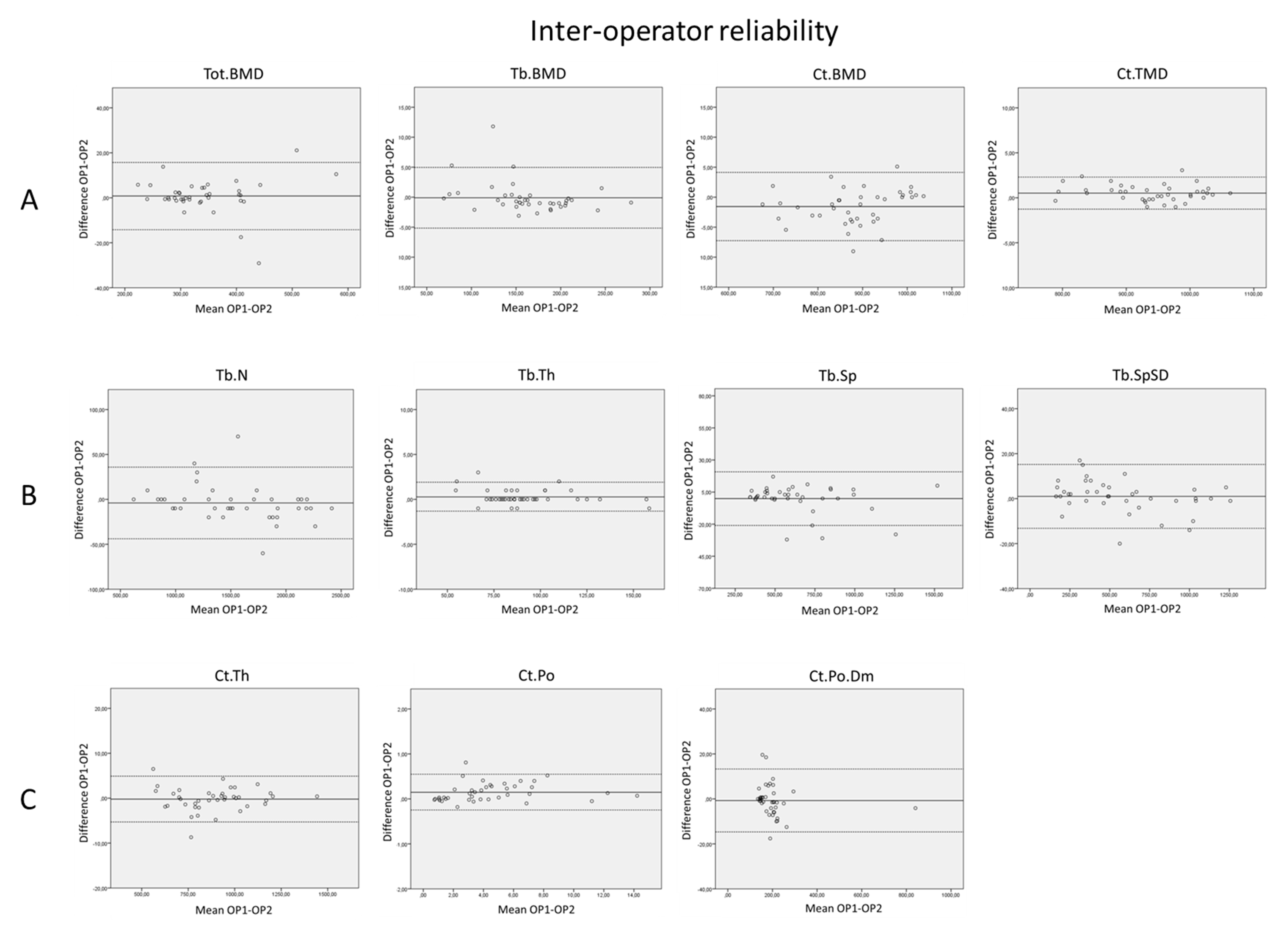

Supplement: Supplementary file 3 — Bland-Altman plots of the inter-operator reliability for the bone density and bone micro-structural parameters. Bland-Altman plots for all bone density (A), trabecular micro-structure (B) and cortical micro-structure (C) parameters for the inter-operator reliability. For all parameters, no cut-off bias was observed and the errors were independent of the mean values detected. BMD, volumetric bone mineral density; Tot.BMD, total BMD; Tb.BMD, trabecular BMD; Ct.BMD, cortical BMD; Ct.TMD, cortical bone tissue BMD; Tb.N, trabecular number; Tb.Th, trabecular thickness; Tb.Sp, trabecular separation; Tb.SpSD, intra-individual distribution of trabecular separation; Ct.Th, cortical thickness; Ct.Po, cortical porosity; Ct.Po.Dm, cortical porosity diameter (TIF 950 kb) [file 12880_2018_255_MOESM3_ESM.tif]

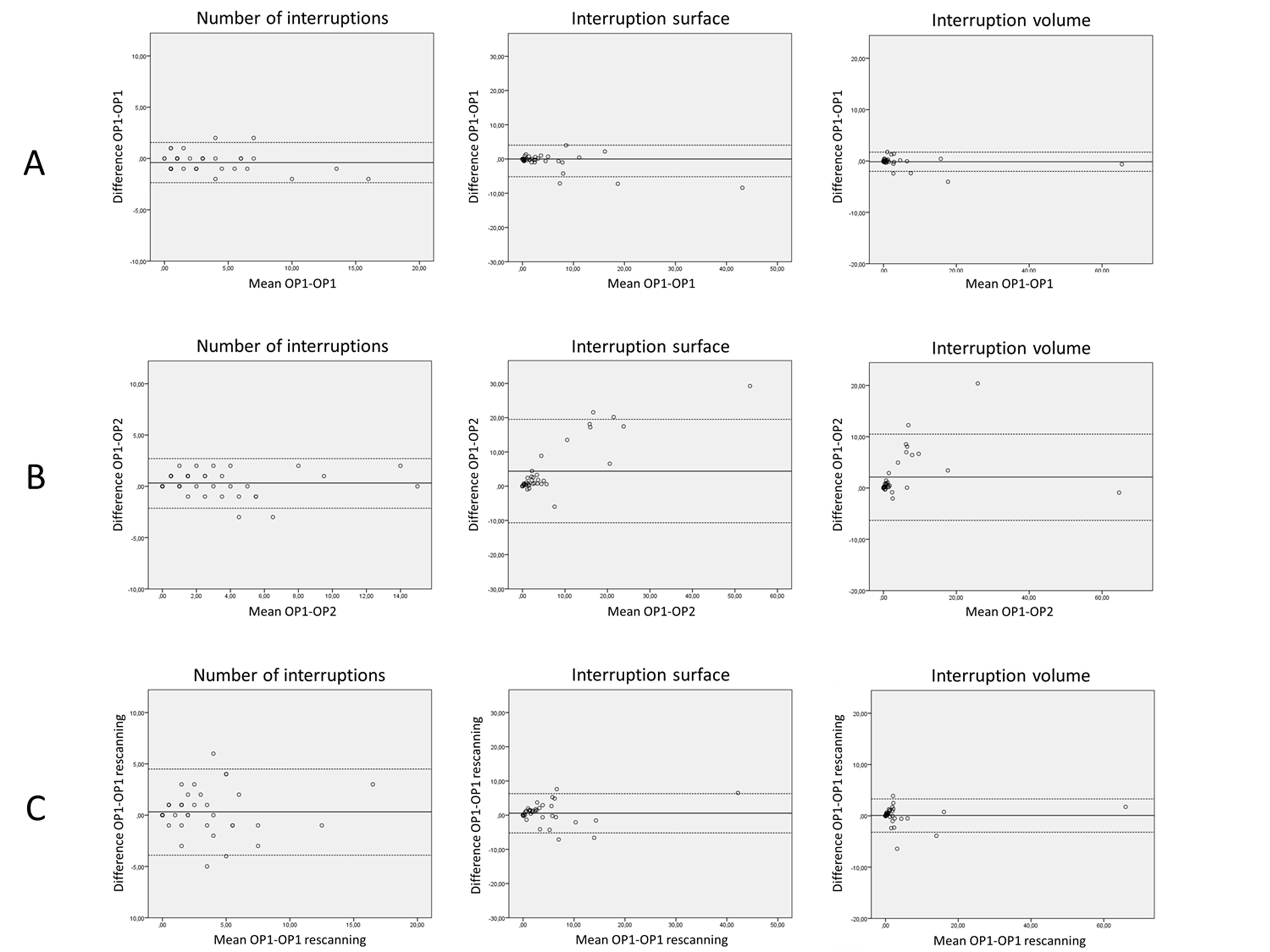

Supplement: Supplementary file 5 — Bland-Altman plots of the intra- and inter-operator reliability and intra-operator reproducibility for all cortical interruption parameters. Bland-Altman plots for all cortical interruption parameters for the intra- operator reliability (A), inter-operator reliability (B) and intra-operator reproducibility (C). (A) For the intra-operator reliability, no cut-off bias was observed for the number, surface and volume of interruptions and the errors were independent of the mean values detected. (B) For the inter-operator reliability, no cut-off bias was observed for the number of interruptions and the errors were independent of the number of interruptions detected. For the interruption surface and volume, OP2 had higher outcomes compared to operator 1, and this increased with increasing mean value. (C) For the intra-operator reproducibility, no cut-off bias was observed for the number, surface and volume of interruptions and the errors were independent of the mean values detected (TIF 971 kb) [file 12880_2018_255_MOESM5_ESM.tif]

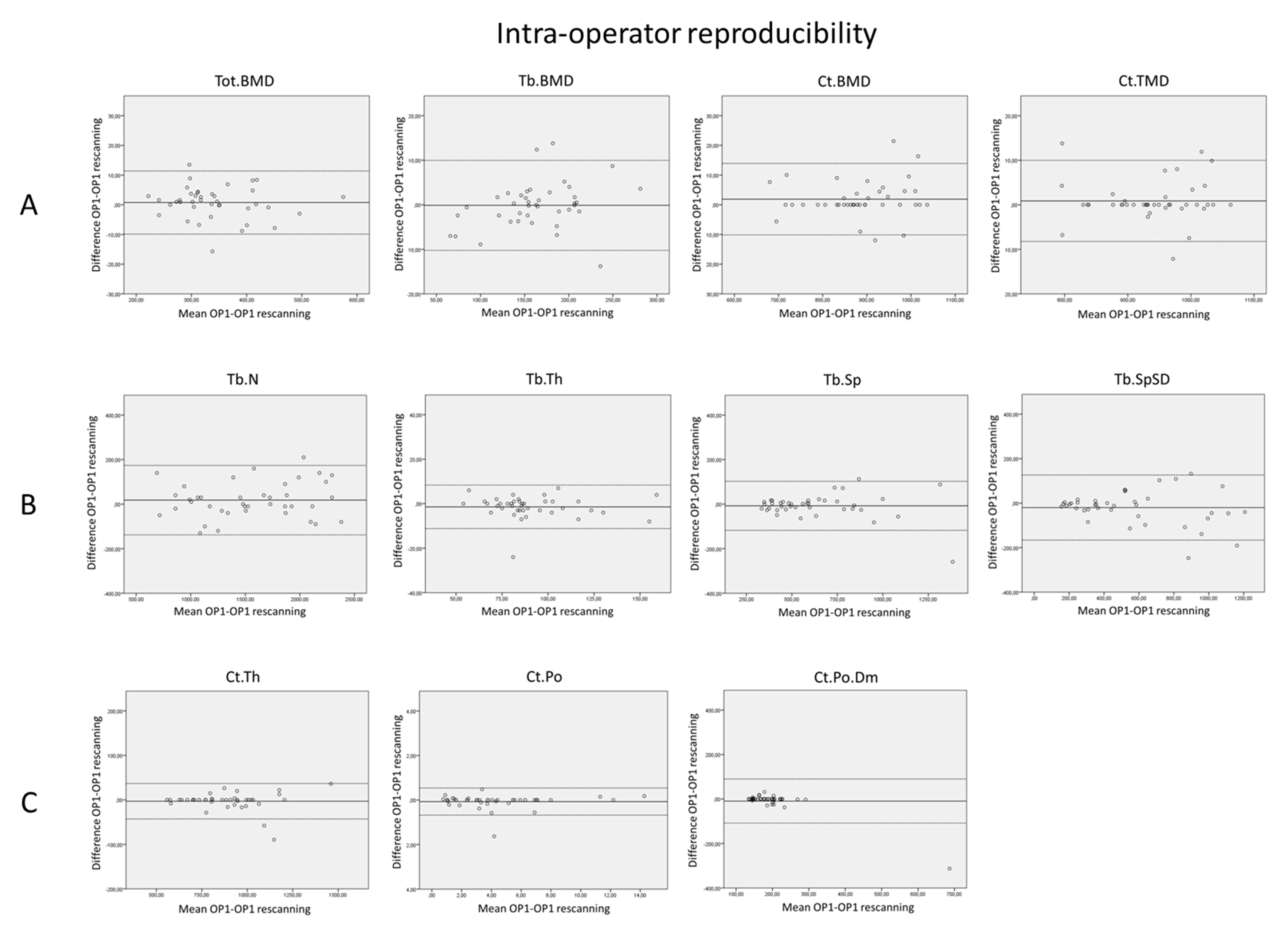

Supplement: Supplementary file 6 — Bland-Altman plots of the intra-operator reproducibility for the bone density and bone micro-structural parameters. Bland-Altman plots for all bone density (A), trabecular micro-structure (B) and cortical micro-structure (C) parameters for the intra-operator reproducibility. For all parameters, no cut-off bias was observed and the errors were independent of the mean values detected. BMD, volumetric bone mineral density; Tot.BMD, total BMD; Tb.BMD, trabecular BMD; Ct.BMD, cortical BMD; Ct.TMD, cortical bone tissue BMD; Tb.N, trabecular number; Tb.Th, trabecular thickness; Tb.Sp, trabecular separation; Tb.SpSD, intra-individual distribution of trabecular separation; Ct.Th, cortical thickness; Ct.Po, cortical porosity; Ct.Po.Dm, cortical porosity diameter (TIF 1028 kb) [file 12880_2018_255_MOESM6_ESM.tif]
