# Supplementary material for: An automated algorithm for the detection of cortical interruptions and its underlying loss of trabecular bone; a reproducibility study
Source: BMC Med Imaging. 2018 May 15;18:13. doi: 10.1186/s12880-018-0255-7 (PMC5952860; doi:10.1186/s12880-018-0255-7)
Supplement: Supplementary file 4 — Intra- and inter-operator reliability of the cortical interruption parameters. Table with the intra- and inter-operator reliability of the cortical interruption parameters. Values are as value (95% Confidence Interval) ICC, intra-class correlation coefficient; SDRMS, root mean square of the standard deviation; LSCSD, absolute least significant change (DOCX 20 kb) [file 12880_2018_255_MOESM4_ESM.docx]

_Supporting Table 1. Intra- and inter-operator reliability of the cortical interruption parameters._

|  |  |  | Intra-operator reliability | | |  | Inter-operator reliability |
| --- | --- | --- | --- | --- | --- | --- | --- |
|  |  |  | ICC  (95%CI) | SD_RMS_ | LSC_SD_ |  | ICC  (95%CI) |
| Number of interruptions |  |  | 0.96  (0.92 – 0.98) | 0.7 | 2.0 |  | 0.94  (0.89 – 0.97) |
| Interruption surface | mm^2^ |  | 0.95  (0.92 – 0.98) | 1.7 | 4.6 |  | 0.70  (0.41 – 0.84) |
| Interruption volume | mm^3^ |  | 1.00  (0.99 – 1.00) | 0.7 | 1.9 |  | 0.91  (0.81 – 0.96) |

_Values are as value (95% Confidence Interval)_

_ICC, intra-class correlation coefficient; SDRMS, root mean square of the standard deviation; LSCSD, absolute least significant change;_

_Table 1. Reproducibility of the cortical interruption parameters, and bone density and structure parameters._

|  | ICC (95% CI) | SD_RMS_ | LSC_SD_ | CV_RMS_ | | LSC_CV%_ | ICC (95% CI) | |
| --- | --- | --- | --- | --- | --- | --- | --- | --- |
| Cortical interruption  parameters | |  |  | |  | |  |  |
| Number of interruptions | 0.96  (0.92 – 0.98) | 0.7 | 2.0 |  | |  | 0.94  (0.89 – 0.97) | |
| Interruption surface | 0.95  (0.92 – 0.98) | 1.7 | 4.6 |  | |  | 0.70  (0.41 – 0.84) | |
| Interruption volume | 1.00  (0.99 – 1.00) | 0.7 | 1.9 |  | |  | 0.91  (0.81 – 0.96) | |
| Bone density  parameters | |  |  | |  | |  |  |
| Tot.BMD | 0.99  (0.99 - 1.00) |  |  |  | |  | 1.00  (0.99 – 1.00) | |
| Tb.BMD | 0.99  (0.99 - 1.00) |  |  |  | |  | 1.00  (1.00 – 1.00) | |
| Ct.BMD | 1.00  (1.00 – 1.00) |  |  |  | |  | 1.00  (1.00 – 1.00) | |
| Ct.TMD | 1.00  (1.00 – 1.00) |  |  |  | |  | 1.00  (1.00 – 1.00) | |
| Bone structure  parameters | |  |  | |  | |  |  |
| Tb.N | 1.00  (1.00 – 1.00) |  |  |  | |  | 1.00  (1.00 – 1.00) | |
| Tb.Th | 1.00  (1.00 – 1.00) |  |  |  | |  | 1.00  (1.00 – 1.00) | |
| Tb.Sp | 1.00  (1.00 – 1.00) |  |  |  | |  | 1.00  (1.00 – 1.00) | |
| Tb.SpSD | 1.00  (1.00 – 1.00) |  |  |  | |  | 1.00  (1.00 – 1.00) | |
| Ct.Th | 1.00  (1.00 – 1.00) |  |  |  | |  | 1.00  (1.00 – 1.00) | |
| Ct.Po | 0.99  (0.98 – 1.00) |  |  |  | |  | 1.00  (0.99 – 1.00) | |
| Ct.Po.Dm | 0.99  (0.99 – 1.00) |  |  |  | |  | 1.00  (1.00 – 1.00) | |

_Values are displayed as mean (SD), and for ICC as value (95% Confidence Interval)_

_n.a., not applicable; CVRMS and LSCCV% were only determined for continuous variables_

_ICC, intra-class correlation coefficient; SDRMS, root mean square of the standard deviation; LSCSD, absolute least significant change; CVRMS root mean square of the coefficient of variation; LSCCV% , least significant change in percentages; Tot.BMD, total volumetric bone mineral density; Tb.BMD, trabecular BMD; Ct.BMD, cortical BMD; Ct.TMD, cortical bone tissue BMD; Tb.N, trabecular number; Tb.Th, trabecular thickness; Tb.Sp, trabecular separation; Tb.SpSD, intra-individual distribution of trabecular separation; Ct.Th, cortical thickness; Ct.Po, cortical porosity; Ct.Po.Dm, cortical porosity diameter_
